# Precision oncology with selective RET inhibitor selpercatinib in *RET*-rearranged cancers

**DOI:** 10.1177/17588359231177015

**Published:** 2023-06-21

**Authors:** Mohamed A. Gouda, Vivek Subbiah

**Affiliations:** Department of Investigational Cancer Therapeutics, The University of Texas MD Anderson Cancer Center. Houston, TX, USA; Sarah Cannon Research Institute, 1100 Dr. Martin L. King Jr. Blvd. Suite 800. Nashville, TN 37203, USA

**Keywords:** LOXO292, precision oncology, *RET*, selpercatinib, targeted therapy

## Abstract

Rearranged during transfection (*RET*) is a protooncogene that encodes for receptor tyrosine kinase with downstream effects on multiple cellular pathways. Activating *RET alterations* can occur and lead to uncontrolled cellular proliferation as a hallmark of cancer development. Oncogenic *RET* fusions are present in nearly 2% of patients with non-small cell lung cancer (NSCLC), 10–20% of patients with thyroid cancer, and <1% across the pan-cancer spectrum. In addition, *RET* mutations are drivers in 60% of sporadic medullary thyroid cancers and 99% of hereditary thyroid cancers. The discovery, rapid clinical translation, and trials leading to FDA approvals of selective *RET* inhibitors, selpercatinib and pralsetinib, have revolutionized the field of *RET* precision therapy. In this article, we review the current status on the use of the selective *RET* inhibitor, selpercatinib, in *RET* fusion-positive tumors: NSCLC, thyroid cancers, and the more recent tissue-agnostic activity leading to FDA approval.

## RET biology

The rearranged during transfection (*RET*) gene is a protooncogene that is located on chromosome 10. It encodes for a receptor tyrosine kinase that initiates a cellular signaling cascade leading to cell proliferation and growth. The RET receptor is composed of three distinct parts: an extracellular domain, a transmembrane domain, and an intracellular domain. The extracellular domain includes four cadherin-like domains, a calcium-binding site, and a cysteine-rich region. The intracellular domain features a tyrosine kinase enzyme, which can have variable isoforms of the c-terminal tail due to alternative splicing.^1–4^ Ligand binding to the RET co-receptors leads to the activation of multiple downstream cellular signaling pathways including RAS/MAPK/ERK, PI3K/AKT, and JAK/STAT; all with resulting increase in cellular proliferation and differentiation.^5–9^

## *RET* fusions

**Figure 1.**
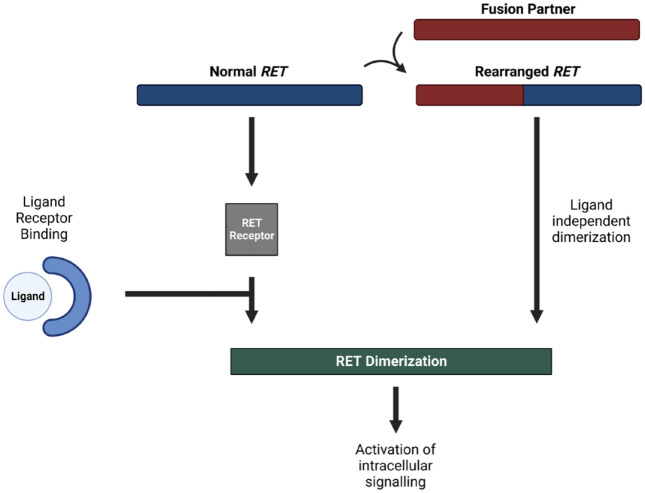
RET fusions can lead to ligand-independent activation of the RET pathway, which leads to downstream signaling of multiple other cellular pathways associated with cellular proliferation and survival. RET, Rearranged during Transfection.

*RET* can be aberrantly activated by mutations and chromosomal rearrangements (Figure 1); both of which has been linked to the process of oncogenesis in different tumor types.^2^ Initial discoveries were made in patients with thyroid cancer who had multiple endocrine neoplasia syndrome, but later evidence suggested a role of *RET* alterations in other sporadic cancers as well.^10–14^*RET* mutations are relatively more frequent, but *RET* fusion-positive cancers represent a distinct molecular entity that defines a unique clinical subtype.^15,16^

In a study including 96,324 samples from AACR Project GENIE, 223 *RET* fusions (0.23%) were identified. Nearly half of *RET* fusions (54.3%) were identified in patients with non-small cell lung cancer (NSCLC). The second most common tumor type with frequent *RET* fusions was papillary thyroid cancer (22.8%). Frequently encountered fusion partners were *KIF5B, CCDC6*, and *NCOA4* .^16^

In disease-specific analysis, *RET* fusions are estimated to occur in 2% of NSCLC patients.^15,17–22^ Such prevalence might be perceived as infrequent, but the fact that lung cancer is estimated to hit nearly a quarter million new patients a year in the United States alone makes the number of patients who might benefit from targeted treatment substantial.^23^*RET* fusion-positive cancers usually present with distinct clinicopathological characteristics including young age, never smokers, early nodal metastasis, and poorly differentiated histology.^15^ A study by Drilon *et al.*^24^ also suggested that *RET*-rearranged lung cancers commonly present with brain metastasis (present in 25% of patients with stage IV at the time of diagnosis with a lifetime prevalence of 46%) and have suboptimal response to multikinase inhibitor (MKI) therapy. In NSCLC samples with *RET* fusion, co-occurring alterations were found in *KRAS, SETD2, PBRL4, EZH1*, and *RRAGC* genes.^16^ In addition to NSCLC, *RET* fusions have also been implied as part of the molecular profile in various other tumor types.^17^

## Detection of RET fusions

There are multiple methods that can be used to detect *RET* fusions which vary in their advantages and disadvantages.^25^ For example, immunohistochemistry has been long used as a cheap technology for the detection of *RET* aberrations but is limited by its low sensitivity and specificity.^15,19,20,26,27^ Fluorescence in situ hybridization (FISH) can be used to achieve higher sensitivity and specificity, but it cannot identify fusion partners unless the specific fusion partner probe is used.^15,26^ Polymerase Chain Reaction (PCR) is another alternative that can inform about the exact fusion partner, but it can only evaluate specimens based on known molecular profile which is used to select the used primers and limits its ability to discover new or unknown partners.^19,26,28–31^

Therefore, next-generation sequencing emerges as the optimum tool for the detection of *RET* fusion variants, given its high sensitivity and specificity as well as its ability to overcome most of the previously mentioned limits. The cost will remain a challenging concern, especially in low-resource settings but it will hopefully be cheaper with wider applications of genomic testing and more advances in technologies that will characterize the era of personalized cancer medicine.^3,31^

One promising approach is the use of liquid biopsy for the detection of *RET* fusions.^25^ This has gained lots of interest in the past decade given its minimally invasive nature. In a study by Rich *et al.*,^32^ analysis of cell-free DNA (cfDNA) from 32,989 samples collected from patients with diverse cancers revealed the presence of 176 *RET* alterations (mostly fusions) in 170 patients (0.5%). In NSCLC, this is particularly important given the challenges of obtaining repeated tissue samples. Liquid biopsy in that setting can allow for the detection of originally present *RET* fusions at baseline samples and emerging fusions during longitudinal monitoring, which offers patients a chance for real-time assessment of therapeutic targetability in an era with the expanded availability of targeted therapy.^33,34^

## Development of RET inhibitors: A historical perspective

Treatment of *RET*-altered cancers has been quite challenging since response rates to chemotherapy were relatively low. Moreover, limited response and progression-free survival (PFS) benefit has been shown with immunotherapy, possibly due to low levels of Programmed Death Ligand 1 (PDL1) expression and low mutation burden.^35^ The first potential for targeting *RET* alterations came historically from studies that were done on MKIs.^2^ Cabozantinib and vandetanib have emerged, among other MKIs, in that regard as key players with evidence of their activity in *RET*-altered cancers. For example, an objective response rate (ORR) of 28% was observed with cabozantinib in patients with previously treated *RET* fusion-positive NSCLC.^36^ Vandetanib has also demonstrated an ORR of 17% in a similar patient population. Nevertheless, the wide spectrum of toxicities primarily attributed to nonselective inhibition of tyrosine kinases including non-target ones was quite devastating. Moreover, the durability of the response was also another concern.^2,37^

With that in mind, further efforts have led to the introduction of more selective RET-targeting agents.^38,39^ So far, two agents, selpercatinib and pralsetinib, have shown promising results in treating *RET*-driven cancers. Data from clinical trials suggested a potential for both drugs in *RET* fusion-positive cancers and led to their inclusion in standard of care treatment guidelines^40^ (Table 1). This review will primarily focus on selpercatinib and its activity in *RET* fusion-positive cancers starting with NSCLC and expanding beyond that to tissue-agnostic activity.

**Table 1.** Summary of data on FDA and EMA-approved selective RET inhibitors in *RET* fusion-positive solid tumors.

Drug	Clinical trial	FDA indication	EMA indication	Data
Selpercatinib	LIBRETTO-001, NCT03157128	Adult patients with locally advanced or metastatic solid tumors with a *RET* gene fusion that have progressed on or following prior systemic treatment or who have no satisfactory alternative treatment options		ORR = 43.9%^41^
		Adult patients with locally advanced or metastatic NSCLC with a *RET* gene fusion	Advanced *RET* fusion-positive NSCLC not previously treated with a RET inhibitor	ORR = 84% and 61% in untreated and previously treated patients^42,43^
		Adult and pediatric patients 12 years of age and older with advanced or metastatic thyroid cancer with a *RET* gene fusion who require systemic therapy and who are radioactive iodine-refractory (if radioactive iodine is appropriate)	Advanced *RET* fusion-positive thyroid cancer who require systemic therapy following prior treatment with sorafenib and/or levatinib	ORR = 79%^44^
Pralsetinib	ARROW, NCT03037385	Adult patients with metastatic RET fusion-positive NSCLC	Adult patients with *RET* fusion-positive advanced NSCLC not previously treated with a RET inhibitor	ORR = 70% and 61% in untreated and previously treated patients^45^
		Adult and pediatric patients 12 years of age and older with advanced or metastatic *RET* fusion-positive thyroid cancer who require systemic therapy and who are radioactive iodine-refractory (if radioactive iodine is appropriate)		ORR = 89%^46^

EMA = European Medicines Agency; FDA = Food and Drug Administration; NSCLC, non-small cell lung cancer; ORR, objective response rate; RET, Rearranged during Transfection.

## Selpercatinib

### Mechanism of action and preclinical data

Selpercatinib is a selective small molecule inhibitor of RET kinase via ATP competitive mechanism. Preclinical studies have shown that selpercatinib possesses high selective potency against different *RET* alterations, including fusions and mutations.^47,48^

### Clinical development in NSCLC

Evidence in favor of using selpercatinib in *RET* fusion-positive NSCLC came from the LIBRETTO-001 trial. LIBRETTO-001 was an open-label phase 1–2 clinical trial including patients with advanced or metastatic solid tumors who harbor *RET* alterations (fusions and mutations). Patients in the phase 2 portion received 160 mg twice daily and were allowed to continue treatment beyond progression per investigator’s evaluation of clinical benefit.

A total of 247 patients with heavily pretreated and 69 patients with treatment naïve *RET* fusion-positive NSCLC were included as part of LIBRETTO-001. ORR was 61% (95% Cl: 55–67) in pretreated patients—including 18 patients with complete response, and 84% (95% CI: 73–92) in previously untreated patients – including four patients with complete response. The median PFS was 24.9 months (95% CI: 19.3–not reached) and 22 months (95% CI: 13.8–not reached) in previously treated and previously untreated patients, respectively.^42,43^ Intracranial activity was quite impressive in 22 patients with measurable central nervous system (CNS) metastasis who showed an ORR of 82% (95% CI: 60–95)—including 23% complete responses. In 80 patients with NSCLC and intracranial disease, the median intracranial PFS was 13.7 months (95% CI: 10.9–not reached).^49^ In an updated analysis including 106 patients with baseline intracranial disease, intracranial ORR was 85% (95% CI: 65–96) with a median PFS of 19.4 months (95% CI: 13.8–not reached). The calculated probability of CNS progression in brain metastasis-free patients who received selpercatinib was only 0.7% at 2 years.^43^ Based on results from the NSCLC cohort analysis in LIBRETTO-001, selpercatinib received its FDA approval for treatment of metastatic *RET* fusion-positive NSCLC in 2020.^50^

Since LIBRETTO-001 was a single-arm study, an effort to explore the comparative effectiveness of selpercatinib by pooling patient-level data from matched patients in real world, pemetrexed/platinum arm of the KEYNOTE-189 trial, and docetaxel arm of REVEAL trial. PFS was significantly longer for selpercatinib (median not reached) *versus* pemetrexed and platinum in KEYNOTE-189 (median 12 months) and docetaxel (median 9 months) using targeted maximum likelihood estimation.^51^

Selpercatinib maintained its efficacy in NSCLC and tolerable safety profile when tested in different patient populations and different disease settings. For example, in a population with Japanese patients (*n* = 44 previously treated and 4 previously untreated), the ORR was 55.4%. Another study (LIBRETTO-321; NCT04280081) included Chinese patients with *RET*-altered cancers. In 47 patients with *RET* fusion-positive NSCLC, ORR was 69.2% (95% CI: 48.2–85.7).^52^ Beyond clinical trials, in a real-world retrospective study, selpercatinib was demonstrated to achieve an ORR of 68% and a disease control rate of 92% in 50 patients with RET fusion-positive NSCLC. This was quite interesting given the inclusion of 14 patients (28%) who had a performance status of ⩾2 who would classically be excluded from clinical trials.^53^

### Clinical development beyond NSCLC

**Figure 2.**
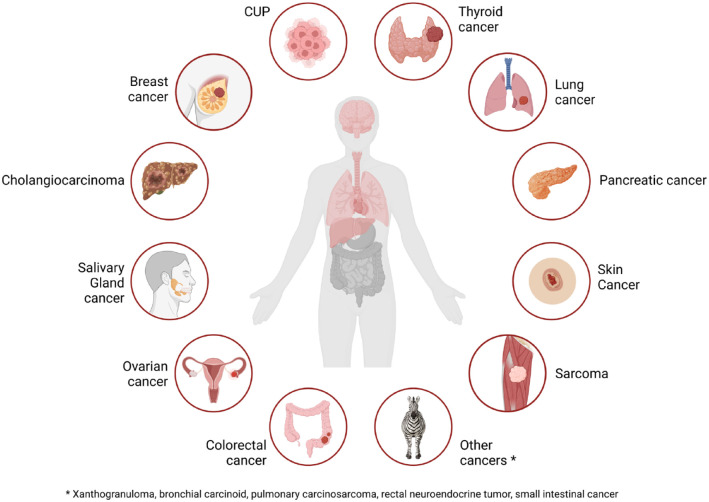
Pan-cancer efficacy of selpercatinib in RET fusion-positive solid tumors. RET, Rearranged during Transfection.

In addition to NSCLC, the initial FDA approval for selpercatinib included patients with advanced or metastatic *RET* mutant medullary thyroid carcinoma and patients with advanced or metastatic *RET* fusion-positive thyroid cancer; based on reports with promising results in those other two other cohorts of LIBRETTO-001.^44^ For example, the *RET* fusion-positive thyroid cancer group showed an ORR of 79% (95% CI: 54–94).^44^ This cohort had patients with variable thyroid cancer histologies including papillary, poorly differentiated, hurthle cell, and anaplastic carcinomas.^44^ Interestingly, selpercatinib use has been demonstrated to enhance radioactive iodine uptake in *RET*-rearranged thyroid cancer, probably via a drug-induced histological redifferentiation.^54,55^ An updated report was published for other cohorts of LIBRETTO-001 and was the basis for the tissue-agnostic approval in 2022.^50^ In 45 patients with RET fusion-positive non-lung and non-thyroid cancers (12 pancreatic cancer, 10 colon cancer, 4 salivary gland cancer, 3 sarcoma, 3 cancer of unknown primary, 2 breast cancer, 2 skin cancer, 2 cholangiocarcinoma, 2 xanthogranuloma, 1 carcinoid syndrome, 1 ovarian cancer, 1 pulmonary carcinosarcoma, 1 rectal neuroendocrine tumor, and 1 small intestinal cancer), the ORR was 43.9% (95% CI: 28.5–60.3)—including two patients with complete response (Figure 2). The median PFS assessed by independent reviewers was 13.2 months (95% CI: 7.4–26.2).^41^

### Drug-induced toxicities

Despite having a tolerable toxicity profile, the use of selpercatinib has been linked to the occurrence of multiple toxicities that can be quite distinct. For example, chylous effusions have been described in patients treated with selpercatinib.^56^ Hypersensitivity reactions have also been reported in selpercatinib-treated patients regardless of prior use of immunotherapy.^57^ Other common adverse events include fatigue, hypertension, rash, dry mouth, nausea, abdominal pain, diarrhea, constipation, edema, and headache.^50^

### Resistance to selpercatinib

Multiple mechanisms of acquired resistance, which commonly limits the durability of response with tyrosine kinase inhibitors, are also being increasingly reported with selpercatinib. While selpercatinib can structurally evade the gatekeeper mutations of *RET* by wrapping around the tyrosine kinase,^58^ resistance to first-generation RET inhibitors, including selpercatinib, has been reported to occur as a result of acquired mutation at the non-gatekeeper sites; namely, solvent front and hinge sites of RET kinase; including *RET Y806* and *RET G810* mutations.^58,59^ These form the basis for the design of second-generation RET inhibitors. For example, Solomon et al. demonstrated using cfDNA samples from a patient with *CCDC6–RET* NSCLC with prior dramatic response to selpercatinib the emergence of *RET G810C* mutation at the time of progression.^59,60^ In addition to *G810* mutations, other *RET*-independent resistance mechanisms have also been reported in *RET* inhibitor-treated patients including amplifications of *MET* and *KRAS* genes.^61,62^*NTRK3* fusion as a mechanism of resistance has also been reported in *RET* fusion-positive lung cancer.^63^

Different approaches have been suggested to overcome such resistance including combination with other targeted agents, for example, crizotinib.^61^ Moreover, second-generation drugs are currently being explored in early-phase trials and will hopefully delay the emergence of these mutations with a benefit in expanding PFS.

### Clinical trials with selpercatinib in multiple settings and future perspectives

The tissue-agnostic approval of selpercatinib was a landmark in biomarker-driven precision oncology. However, multiple studies are currently ongoing to explore the expanded potential of selpercatinib in *RET* fusion-positive cancers (Table 2). These are primarily focused on testing in different disease settings and patients’ populations. For example, a phase 3 trial (LIBRETTO-432; NCT04819100) is investigating the use of selpercatinib in the adjuvant setting compared to placebo when given to patients with early-stage NSCLC after curative intent surgery or radiation therapy.^64^ In the neoadjuvant setting, NCT04759911 is a phase 2 trial that is evaluating preoperative selpercatinib in patients with thyroid cancer and RET alterations.^65^

**Table 2.** Examples of ongoing clinical trials for selpercatinib in *RET* fusion-positive cancers.

Clinical trial	Phase	Setting	Population
LIBRETTO-432	3	Adjuvant	Patients with early-stage NSCLC after curative intent surgery or radiation therapy
NCT04759911	2	Neoadjuvant	Patients with thyroid cancer
LIBRETTO-431	3	Advanced	Patients with advanced or metastatic *RET* fusion-positive nonsquamous NSCLC
Lung-MAP	2	Advanced	Patients with *RET* fusion-positive recurrent or metastatic NSCLC
ORCHARD	2	Advanced	Patients with advanced NSCLC who progressed after treatment with first-line osimertinib
FINPROVE	2	Advanced	Patients with advanced solid tumors that harbor a *RET* alteration
LIBRETTO-121	1/2	Advanced	Pediatric patients with advanced solid tumors and primary CNS tumors not including lung cancer that harbor a *RET* alteration
Pediatric-MATCH	2	Advanced	Pediatric patients with *RET*-altered cancers

NSCLC, non-small cell lung cancer; *RET*, Rearranged during Transfection.

In the advanced and metastatic setting, LIBRETTO-431 (NCT04194944) continues to evaluate the efficacy of selpercatinib in patients with advanced or metastatic *RET* fusion-positive non-squamous NSCLC. Patients are randomized to receive either selpercatinib or standard platinum-based and pemetrexed-based therapy with or without pembrolizumab as first-line treatment.^60^ Selpercatinib is also tested as part of the Lung-MAP lung cancer Master Protocol which is an umbrella trial that includes patients with advanced NSCLC for the purpose of testing various therapeutic regimens including selpercatinib. For example, phase 2 Lung-MAP (NCT05364645) investigates carboplatin and pemetrexed with or without selpercatinib in patients with RET fusion-positive recurrent or metastatic NSCLC. Another arm of Lung-MAP evaluates selpercatinib as a single-agent in the same disease setting.^66^ Selpercatinib is also being studied as part of the phase 2 platform study (ORCHARD; NCT03944772) in patients with advanced NSCLC who progressed after treatment with first-line osimertinib.^67^ This is also the case in the phase 2 Finnish trial (FINPROVE) which includes patients with advanced solid tumors that harbor a *RET* alteration.^68^

In the pediatric patient population, LIBRETTO-121 (NCT03899792) is a phase 1/2 trial evaluating selpercatinib in patients with advanced solid tumors and primary CNS tumors, not including lung cancer, that harbors a *RET* alteration. Moreover, the phase 2 pediatric MATCH trial (NCT04320888; NCT03155620) is studying selpercatinib in *RET*-altered cancers in the pediatric patient population (⩽21 years).

## Conclusion

Selpercatinib has led to a paradigm change in the management of *RET* fusion-positive solid tumors including NSCLC and thyroid cancer. Its current tissue-agnostic approval highlights the potential it has in different tumor types. Multiple studies are ongoing with the aim of exploring selpercatinib use in other disease settings and different patients’ populations.

## Declarations

**Ethics approval and consent to participate:** Not applicable.

**Consent for publication:** Not applicable.

**Availability of data and materials:** Not applicable.
